# Dermatology and Syphilis

**Published:** 1876-06

**Authors:** 


### IV. Dermatology and Syphilis.

1. *Cerebral Syphilis, with Coma.* Mercier. (*Archives Génér. de Méd.*, May, 1876.)

A large, well-formed man, 33 years old, with little facial color, closed eyes, no œdema of limbs nor emaciation, had 28 respirations to the minute, no cough and a stertorous respiration, due to mucus in the nasal and laryngeal fossæ, completely masking the vesicular murmur. Pulse, 62. Heart-sounds normal. Temp., 37.9° C.

The tongue was moist; belly supple and painless on pressure; urine neither albuminous nor saccharine; scanty, yellowish, diarrhœic, stools—bladder and rectum contents voided without consciousness.

The patient could be roused from this comatose condition only with difficulty. He could then understand questions and occasionally answered them in a few words. He complained of supra-orbital headache. He could not see those surrounding him. His pupils were dilated with slight irregularity, and feebly contracted under the influence of a strong light. The eyes were expressionless. He continually ground his teeth and could scarcely protrude the tongue, but swallowed the soup put into his mouth, some portion returning by the nose. There was also occasional pharyngeal cough with mucous expectoration. There was no paralysis, but no voluntary movement. There was some appreciation of pain, but he seemed indifferent to cold, heat and tickling.

Two small eschars surrounded by an erythematous patch were visible on the buttocks.

There was no delirium, hallucination, contracture, nor convulsion.

Previous health had been excellent, prior to six weeks before examination (date of onset), without scrofulous or rheumatic antecedents. Since the inauguration of the illness his principal symptoms had been : heaviness of the head, loss of power in the limbs, and diminution of visual sense. Cusco, with the ophthalmoscope, had recognized atrophy of the papillæ. Soon after, the coma supervened ; and M. discovered three large rounded cicatrices—superficial, covered with small violaceous crusts.

highly characteristic of syphilis—upon the legs. There was also adenopathy of the neck and groin.

Recovery was complete—even to the restoration of vision—after the prolonged use of calomel and the iodide of potassium. While the improvement was in progress and his lesions of the skin disappearing, he stated that three years before he had suffered from genital chancre, followed in due course by roseola and mucous patches of the mouth, throat and anus. He had also had osteocopic pains.

2. *Cerebral Syphilis.* WOOD. (*Phil. Med. Times*, Feb. 19, 1876.)

Non-specific basal meningitis and basal cerebral tumors are very rare in non-tuberculous adults. Upon this is dependent the fact that slowly developed paralyses of the motor nerves of the eye and its muscles are so generally dependent upon syphilis. Paralysis of the portio dura of the 7th pair, does not have a similar significance for several reasons. The nerve arises so far back and proceeds so immediately outwards to enter the internal auditory meatus, that it is not usually seriously compromised by a basal exudation, and therefore escapes in syphilitic meningitis. On the other hand, owing to its long passage through the bony canal, and to its extremely superficial and exposed point of exit, the facial nerve is excessively apt to be paralyzed by rheumatic or other inflammations and exudations. Hence facial palsy may be said, on the whole, to be antisyphilitic in its expression. It must, however, be borne in mind, that specific palsy of the nerve may occur. I have seen unquestionable cases of it.

3. *Syphilitic Phthisis.* FOURNIER. (*Gazette Hebdom.*, Dec. 17, 1875.)

The author formulates his conclusions as follows:

1. Tertiary syphilis can produce in the lungs, lesions which either locally or by reacting on the general health, simulate pulmonary phthisis.

2. These pulmonary lesions of syphilis are often amenable to specific treatment; however grave and important they may appear, they are far from being beyond the resources of art.

3. Consequently, when a case of pulmonary lesion presents itself, it is important, unless the existence of tuberculosis be quite certain, to ascertain if the lesion can be traced to syphilis. It is necessary always to bear in mind that syphilis is a possible cause f pulmonary lesions.

4. When syphilis can be suspected to be the cause, the primary indication is to prescribe specific treatment, which, in similar cases, has sometimes been followed by the happiest results.

Differential diagnosis:—The syphilitic lesion is unilateral, circumscribed, and without predilection for the pulmonary apices. It generally affects a portion of the lung not more than a few centimetres square, constituting a little islet of disease, surrounded by healthy lung tissue. When the morbid changes are far advanced in this circumscribed spot, the diagnosis of syphilis is pretty safe. The coexistence of a fair degree of *embonpoint* and general health, with advanced pulmonary changes, points to syphilis as the cause; so also does a slow development of the pulmonary lesion, the general condition remaining good. A close examination of the entire body for ordinary symptoms of tuberculosis or syphilis, is of course necessary. Pulmonary syphilis is not transmissible by heredity.

4. *Precocious Malignant Syphilides.* ORY. (*Review of the Lyon Méd.*, Feb. 27, 1876.)

The author discusses three causes of precocity and malignity:—force of the virus, seat of initial lesion, and condition of the contaminated organism. The thirty cases observed occurred — 20 in men and 10 in women.

The author is disposed to believe that malignant syphilis is more rare in women. In fifteen cases there was chronic alcoholism; in thirteen, scrofula, mental disturbances, poverty or the feebleness of convalescence from grave disease and debauchery. These causes were sometimes associated. In some of the cases of alcoholism, the patients, though young and of a healthy aspect, had grave symptoms. Scrofula was only distinctly potent in two cases, where the latter disease was well marked. (Ricord used to say that there was a "scrofulate of pox."—ED.) The influence of poverty, mental depression, dissipation, lactation and pregnancy is evident in the following conclusion :—" That which enfeebles the organism is the cause or a cause of malignant syphilis."

The author differs from Van Swieten and other writers, in believing that extra-genital chancres are not followed by grave forms of disease, but twelve of his cases had malignant syphilis, of which four had chancres of the lip, three of the arm, and one of the chin.

Ory does not believe that the gravity of syphilis depends upon the power of the syphilitic virus. The reviewer combats this idea, and cites Dubuc and others to show that the gravity of syphilis often is due to the disease itself alone. He also criticises the author in his omitting to mention the age of the patient and the phagedenic phases of chancre as determining causes of gravity. The utility of a mercurial treatment even in these forms of syphilis is dwelt upon.

(Neither the author nor his reviewer mentions a cause of malignancy in syphilis which has been forcibly and clearly demonstrated by Fournier, viz., *unrecognized and therefore untreated chancre.* Given a case of syphilis, which under early and persistent treatment results in trifling developments upon the mucous and cutaneous surfaces, can it be doubted that in the absence of all treatment, either from non-recognition of the disease or from other causes (homœopathic folly, a long sea voyage, frontier life, etc.), the malady would certainly develop precociously and very probably become malignant.—ED.)

5. *The Influence of Erysipelas on Syphilis.* DR. A. DEAHNA. (*Viertel-jahresschr. of Derm. and Syph.* III, 1.)

French writers (Ricord, Sabatie, Rayer, *et al.*) have repeatedly mentioned the salutary influence of acute febrile diseases, and especially erysipelas, upon syphilis. In confirmation of this, Dr. D. reports the following observations made at the surgical clinic in Freiburg, Germany:

In Nov., 1873, a laborer, aged 27 years, was admitted to the hospital with a papular syphilide, which pretty uniformly extended over the face, the trunk and the extremities. There was also an induration of the prepuce and a general enlargement of the sympathetic glands. At the same time, the patient suffered from a chronic inflammation of the left elbow-joint with fistulous openings, the left arm being anchylosed in a nearly straight position. When the patient had received four hypodermic injections of sublimate (one-sixth of a grain each) he was given chloroform and his anchylosed arm was bent to a right angle and dressed with a plaster-of-paris bandage. Erysipelas being epidemic in the hospital at that time, the patient was affected by it the day after the operation. Starting from the affected elbow it rapidly extended over the whole arm, the shoulder, the breast and the back. The erysipelas lasted two weeks, and after the first week the induration of the prepuce and the syphilitic eruption of the skin had disappeared entirely. And the patient remained free from any syphilitic symptom until the seventeenth of January, 1875, when a roseola appeared sparsely over the body, avoiding, however, those portions which had been the seat of the erysipelas. From February 4th to 10th he had another attack of erysipelas affecting the left arm. On the third day of its appearance the syphilitic roseola had disappeared, and the patient has never since had any sign of the malady. In March, the diseased elbow was excised and the wound healed as readily as in any non-syphilitic patient. As the mercurial treatment had been too short to be effective, the destruction of the syphilitic virus, the doctor thinks, must be attributed to the influence of the erysipelas.

6. *Effects Produced upon the Blood of Syphilitic Patients by Mercury.* KEYES. (*N. Y. Medical Record*, Dec., 1875.)

This valuable and interesting paper gives the results of a series of experiments made by the author with the aid of the *hématimètre*, in counting the red blood corpuscles of syphilitic and other subjects. We have space only for his conclusions.

1. Average of red blood-corpuscles in one cubic mm. of blood of healthy adult male. A high average is five millions. Anæmia rarely goes below three millions, and in five instances the count reached above six millions.

2. Effect of small doses of mercury upon the blood early in syphilis. In all the cases counted, the number of the red blood-corpuscles increased under the influence of mercury, good hygiene and tonics.

3. Effect of long-continued and of small doses of mercury upon the blood in syphilis. In three cases the drug was administered respectively, eleven, six and eighteen months—the blood count was above the healthy average, and clinically they were all in excellent health.

4. Effects of mercury in excess upon the blood in syphilis. In this (the only case in which salivation had, for special reasons, been induced,)

the count showed a loss of one million, which was attributed to the excessive use of mercury.

5. Effect of mercury combined with the iodides upon the blood in syphilis. In this list it would be fair to expect frequent exceptions to the rule of increase, because so many who need prolonged treatment, late in the disease become more or less cachectic and depreciated in general health, but in only two of the nine cases under this head did the average count fall below the standard, and this among patients who had had syphilis for a long time.

6. Effect of mercury upon the blood in syphilis in hospital cases. Of these cases one entered salivated and his count increased when he began to eat. One showed a wretched count, was debilitated by disease and hospitalism, but improved under the influence of good hygiene and tonics; and it was believed that the mercury also helped him.

7. Effect of small doses of mercury upon the blood of individuals not syphilitic. The observations showed an increase in the count, one-fifth of a grain of the potassic iodide was given in granules after each meal, and one granule was each day added after the fourth day, until there were evidences of irritative action, when the dose was reduced one-half and continued there.

The following are the author's deductions:

1. Mercury decreases the number of the red cells when given in excess, especially in hospital patients.

2. Syphilis diminishes the number of the red cells below the healthy standard.

3. Mercury in small doses, continued for a short or long period in syphilis, given alone or with iodide of potassium, increases the number of red blood-corpuscles and maintains a high standard of the same.

4. Mercury in small doses acts as a tonic upon healthy animals, increasing their weight. In larger doses it is debilitating or fatal.

5. Mercury in small doses is tonic (for a time at least) for individuals in fair health not syphilitic. In such, the number of red corpuscles is increased.

